# Structural Design of Vascular Stents: A Review

**DOI:** 10.3390/mi12070770

**Published:** 2021-06-29

**Authors:** Chen Pan, Yafeng Han, Jiping Lu

**Affiliations:** 1 School of Mechanical Engineering, Beijing Institute of Technology, Zhongguancun South Street No. 5, Haidian District, Beijing 100081, China; 3220195044@bit.edu.cn (C.P.); jipinglu@bit.edu.cn (J.L.); 2 Institute of Engineering Medicine, Beijing Institute of Technology, Zhongguancun South Street No. 5, Haidian District, Beijing 100081, China

**Keywords:** vascular stent, ISR, medical device, bridge, representative volume unit (RVE)/representative unit cell (RUC), patient-specific

## Abstract

Percutaneous Coronary Intervention (PCI) is currently the most conventional and effective method for clinically treating cardiovascular diseases such as atherosclerosis. Stent implantation, as one of the ways of PCI in the treatment of coronary artery diseases, has become a hot spot in scientific research with more and more patients suffering from cardiovascular diseases. However, vascular stent implanted into vessels of patients often causes complications such as In-Stent Restenosis (ISR). The vascular stent is one of the sophisticated medical devices, a reasonable structure of stent can effectively reduce the complications. In this paper, we introduce the evolution, performance evaluation standards, delivery and deployment, and manufacturing methods of vascular stents. Based on a large number of literature pieces, this paper focuses on designing structures of vascular stents in terms of “bridge (or link)” type, representative volume unit (RVE)/representative unit cell (RUC), and patient-specific stent. Finally, this paper gives an outlook on the future development of designing vascular stents.

## 1. Introduction

**Figure 1:**
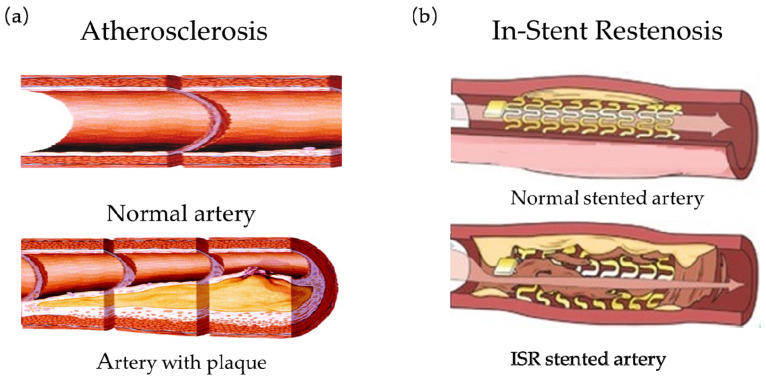
Atherosclerosis of blood vessels and in-stents restenosis: (**a**) Atherosclerosis [1]; (**b**) ISR [7].

Atherosclerosis is one of the cardiovascular diseases. Its pathological mechanism is that fat or lipid substances are deposited on the arterial wall under the influence of various cardiovascular risk factors. These depositions form a large number of plaques, leading to arterial wall thickening, causing a vascular blockage, and affecting blood flowing (as shown in Figure 1a [1]). Severe atherosclerosis can also cause coronary artery disease, stroke, peripheral artery disease, or kidney problems. At present, the most common and effective treatment method in the world is Percutaneous Coronary Intervention (PCI) [2,3]. PCI is to unblock and restore blood by placing a vascular stent on the stenosis and hardening of the artery for expansion. PCI is minimally invasive and highly effective. In the treatment of PCI, the stent is a tiny tubular structure and used to expand the vessel wall and expand the vascular lumen to prevent the artery wall from recoiling and restore the cardiovascular obstructed by atherosclerosis [4,5]. Therefore, the vascular stent, as the sophisticated medical device for clinical treatment, should have ideal functions and mechanical properties [6]: (1) high elasticity to realize the curling and re-expansion of the stent in the blood vessel; (2) high strength and fatigue resistance to withstand the periodic physiological load of arteries; (3) good biocompatibility to reduce the incidence of thrombosis and vascular restenosis and alleviate implant rejection in the body. In addition, there are other properties. In addition to these features mentioned, 13 different properties of ideal stent were listed in the review article of Liu et al. [7], which provides a great help for the design of vascular stents. And Liu et al. also pointed out that there were no perfect stents. The current clinical application of vascular stents, after decades of development, has the corresponding therapeutic function and mechanical properties. Manufacturing technology and surgical technology are gradually becoming mature. What’s more, the arterial blockage after interventional therapy has been significantly reduced. However, each stent still has its own advantages and drawbacks inevitably. A stent cannot cover all ideal properties, and usually offers several good properties. The design structure of stent is related to restenosis. There are still many complications in the current PCI treatment. Among them, In-Stent Restenosis (ISR) is the most common complication (as shown in Figure 1b [8]), which is an important challenge for biomechanical engineering, and has an impact on designing stent. Studies have shown that one year after the implantation of ordinary metal stents, the probability of restenosis at the diseased blood vessel is as high as 20–40% [9], and about 10% of patients need to re-implant the stent. Even for the drug-eluting stent, which has been widely used in recent years, the restenosis rate in the stent after implantation is as high as 8–15% [10,11].

At present, the causes of ISR have not been fully found and discovered. Some scholars believed that it is the result of huge changes in the geometric structure of the artery after the stent is implanted [12], and some scholars hold that the lower shear stress in the artery is also one of the reasons for restenosis [13,14]. The more important reason is that in order to ensure that the vascular stent has sufficient radial stiffness to support the inner wall of the blood vessel, Young’s modulus and hardness of the selected stent material are higher than that of the vessel. Colombo et al. elucidated the possible link between altered hemodynamics and ISR progression [15]. They found that focal re-narrowing frequently occurred after investigating from six months to 12 months. In this section, the factors triggering ISR are the following: (1) different geometry between expanded stent and blood vessel. If the stent does not match the geometry of the diseased blood vessel after expansion, a strong interaction force will generate between the stent and the inner vessel wall resulting in stress concentration [16], which damages the inner wall of the vessel and gives rise to ISR. (2) Structural stability of stent. The degree of blood vessel curvature changes with the movement of the human body, studies have demonstrated that the degree of vascular curvature of the human body is in the range of 30° to 150°, especially the degree of vascular curvature in the carotid artery position, which requires the stent to be well adapted to the geometric shape of the vascular curvature [17]. Studies have indicated that in the blood vessel with a higher degree of curvature, the stent exerted higher stress on the vessel wall and caused greater damage [18]. In a word, the stent has to keep stability after implanting. (3) Compatible mechanical properties of the stent. Due to the influence of structure and material properties, the stent has a tendency to “straighten” during the expansion process, which has a straightening effect on the diseased blood vessel. Therefore, the bending degree of the curved blood vessel would generally be significantly reduced after the stent is implanted [19,20,21]. Gyöngyösi et al. [19] found that before and after the stent was implanted into the aorta, the bending angle of the blood vessel was reduced from 67° to 58°, and the curvature of the blood vessel was significantly reduced. Wu et al. [21] found there were high-stress gradients and stress concentration at both ends of the stent through finite element simulation. Researchers have been looking for stents that can be bent along with blood vessels to treat the diseased blood vessels in high bending parts, such as arches and bifurcations so that the stent can be in good fitness with the curvature of the blood vessel after expansion [22]. Nevertheless, by designing a reasonable stent to make the stent have the same curved shape as the blood vessel after expansion, it will reduce the damage of the stent to the vessel wall and relieve the stress concentration of the blood vessel. Consequently, the structural design of the vascular stent with good mechanical properties and reasonable structures is one of the effective methods to effectively reduce complications such as ISR. Structural design can improve the performances of a vascular stent, in terms of promoting therapeutics, stability, mechanical properties, as well as alleviating side-effects. For instance, various stents should compromise the selection of materials due to specific applications. As a result, the mechanical properties and stabilities might be affected. But structural design can supplement this weakness.

**Figure 2:**
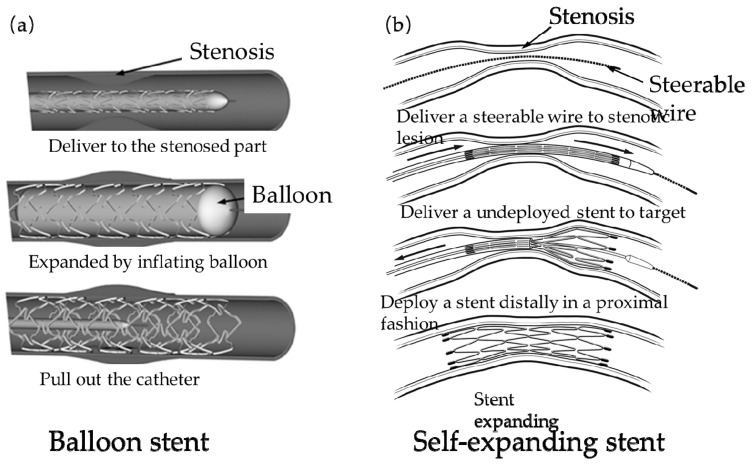
Two expansion forms of vascular stents: (**a**) Balloon stent [23]; (**b**) Self-expanding stent [24].

The way of expansion affects the positioning accuracy and mechanical performance of stents. Currently, the expansion methods of vascular stents implanted in human blood vessels include balloon expansion (Figure 2a [23]) and self-expanding (Figure 2b [24]). Whether it is a balloon-expandable stent or a self-expanding stent, the structure of the stent has a direct impact on the mechanical properties, such as the elastic-plastic stress distribution after the stent is compressed before delivery to the blood vessel, the radial stiffness, and axial flexibility after the stent is expanded, fatigue failure under the influence of periodic blood pressure. A vascular stent implantation is a common operation of interventional cardiologists at present, and the market for its development and design is also expanding and developing. Therefore, in order to solve the problem of in-stent restenosis and treat cardiovascular diseases, it is of great significance to design a stent with multiple mechanical properties from the perspective of the structure of the vascular stent.

Although there have been some reviews about vascular stents [7,25], with the continuous development of stent design, it is very helpful to guide the structural design to supplement and improve review by updating the latest stent structure. This paper aims to summarize the types of vascular stent structure designed in recent decades, make a new classification of the existing stent structure, evaluate the influence of structure on mechanical properties, and predict the future direction of the design of vascular stent, and summarize the progress of the research on the design of vascular stent structure.

## 2. Introduction to the Vascular Stents

### 2.1. The First Generation of Vascular Stents: Bare Metal Stents (BMS)

**Figure 3:**
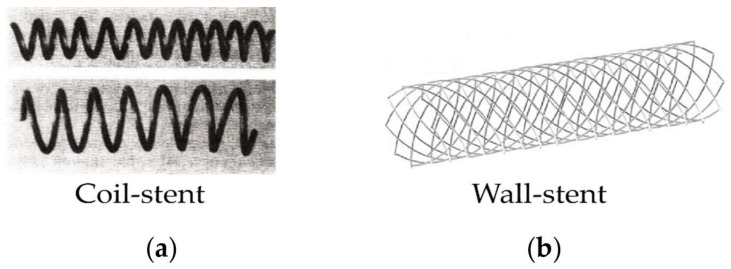
Two structures of braided stents: (**a**) Coil-stent [28]; (**b**) Wall-stent [33].

In 1969, Dotter, using stainless steel to wrap the coil stent, took the lead in researching the structure of vascular stent, and successfully carried out animal implantation experiments [26,27,28]. Figure 3a showed the Nitinol alloy-wrapped vascular stent designed by Dotter [28]. Since the wrapped structure stent is spirally wound with one or more wires, although it has good bending flexibility, its radial stiffness is extremely poor, and the radial force does not support the vessel wall insufficiently, which results in larger elastic recoil of the stent. Research reports showed that the wrapped stent caused the restenosis rate of the blood vessel to be as high as 57% [29]. Besides, the stent with a wrapped structure is not suitable for treating small-diameter blood vessels due to its large diameter, which limits its development. It was not until 1987 that Sigwart et al. [30] first applied BMS to clinically treat coronary artery disease. This marked the first successful clinical application of vascular stents in humans. Subsequently, in 1988, Palmaz et al. [31] also successfully applied the BMS made of stainless steel to clinical applications. In 1989, Günther et al. [32] used a self-expanding Wall-stent to treat iliac-femoral artery stenosis and occlusion. Figure 2b was the Wall-stent model used by Kim et al. [33] to study the effect of stent structure on blood flow. The BMSs have good radial stiffness and prevent serious elastic recoil and reduce the restenosis rate in the stent. However, BMSs in the later stage of implantation in the human body will still cause a higher rate of ISR [34,35], and the restenosis rate is 20–30% [36]. Because BMSs stay in the human body for a long time even permanently, it will also cause vascular inflammation and the risk of atherosclerosis [37]. Nevertheless, with the development of stent design, BMSs, as the first generation of vascular stents, have greatly improved their structures and play an important role in the treatment of coronary artery disease. At present, BMSs are still widely used in clinical practice applications.

### 2.2. The Second Generation of Vascular Stent: Drug Eluting Stent (DES)

**Figure 4:**
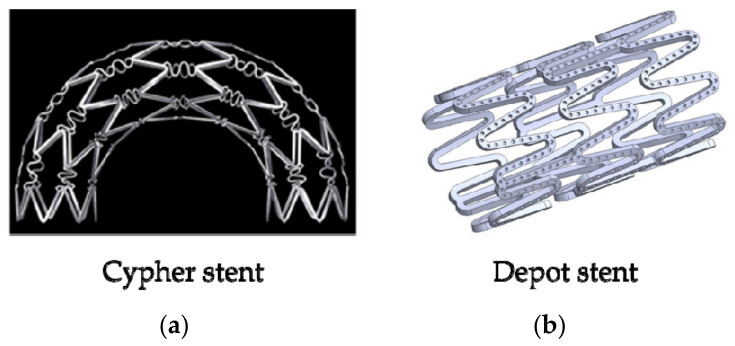
Two structures of DESs: (**a**) Cypher stent [40,41]; (**b**) Depot stent [43].

In order to solve the problem of ISR, the researchers used the bare metal stent as the structural basis and coated it with the biocompatibility coating and anti-proliferative drugs finally developed the second-generation stent—drug-eluting stent (DES), which means bioabsorbable or non-absorbable polymers or polymer-free stents. After renewed research and development, the DESs have been widely used in clinical applications and achieved remarkable therapeutic effects. Currently, eluting drugs include rapamycin, paclitaxel, and everolimus [38], the main substrates are stainless steel, cobalt-chromium alloy, NiTi shape memory alloy, etc. and PLLA (Poly L-lactic acid), PDLLA (Racemic polylactic acid), PCL (Polycaprolactone), PGA (Polyglycolide) and their copolymers are commonly used to prepare DESs [39]. The characteristics of stents are changed due to adding polymers, for example, biocompatibility and biomechanics. There was a detailed introduction of DESs in a review of Karjalainen et al. [40]. They described the coating materials for DESs. In their review, the development of stent materials was introduced more. In 2002, Cordis Corporations took the lead in developing the Cypher structure of DESs, as shown in Figure 4a. Compared with the BMSs, the Cypher stent at the 9th month of implantation, the target lesion revascularization (TLR) was reduced by about 80% and the target vessel revascularization (TVR) was reduced by about 70%. Moreover, the mortality rate and the incidence of myocardial infarction (myocardial infarction, MI) were not significantly different [41,42], and the rate of ISR was 5–10% [43]. DESs can effectively inhibit neointimal hyperplasia, prevent the inflammatory response in the early stage of stent implantation, and significantly improve the rate of vascular restenosis, TLR, and TVR. However, most of the commonly used DESs are permanent metal substrates. After the drug coating on the surface of DESs is decomposed and released, the metal substrates will still remain in the human body permanently, which will cause vascular inflammation and the risk of recurring atherosclerosis in the later stage [37]. In addition, the DESs also have the problem of slow drug decomposition and carrier shedding. Therefore, when designing the structure of the stent, it is necessary to consider designing a structure that can fully decompose the drug and stabilize the carrier. Hsiao et al. [44] designed the drug-eluting stent with a micro-pot structure on the surface (as shown in Figure 4b). The research results demonstrated that the micro-pot structures on the stent surface had an effect on the anti-fatigue performance and drug loading. By changing the size and density of the micro-pot, the drug loading capacity can be controlled, and the structural mechanical properties of the stent can be weighed against the drug loading capacity.

### 2.3. The Third Generation of Vascular Stent: Biodegradable Stent (BDS)

**Figure 5:**
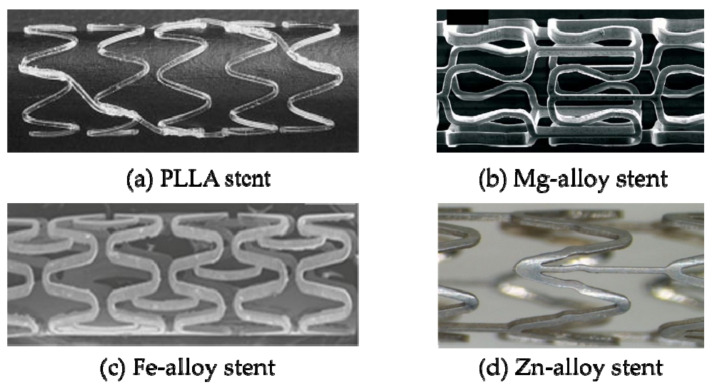
BDSs: (**a**) PLLA stent [52]; (**b**) Mg-allot stent [53]; (**c**) Fe-alloy stent [54]; (**d**) Zn-alloy stent [55].

Since the DESs still have the same problems as the BMSs after the drug layers are released [45]. The ideal solution is to design a stent that has the function of supporting blood vessels in the early stage of implantation. After the diseased vessels return to normal function, the stent can be absorbed or decomposed and eliminated by the body in the later stage to avoid the harm caused by the stent permanently left in the body [46,47]. Biodegradable stent (BDS), as the third-generation stent product, is made of biodegradable or bioabsorbable materials and has good tissue compatibility and biodegradability. After the stent is implanted, the blood vessel can be effectively expanded in the early stage. Finally, the stent can be gradually degraded in the human body. The degradable product can be excreted through metabolism or absorbed by the body without affecting the function of the blood vessel. In 1988, Stack [48] took the lead in developing bioabsorbable stents. In 1991, Stack and Chapman et al. [49] conducted in-depth research on bioabsorbable stents and made animal implantation experiments. They found that within a period of time after implantation, the vascular patency rate was good, there was no inflammation and serious thrombosis. At present, the matrix materials of BDSs mainly include degradable polymers and degradable alloys. The degradable polymers include polylactic acid (PLA), poly-L-lactic acid (PLLA), and polycaprolactone (PCL), racemic polylactic acid (PDLLA), etc. [50], the degradable alloys include magnesium alloys, zinc alloys, and Fe alloys [51]. According to data, the first real application of biodegradable stents in animal experiments was developed by Yamawaki et al. [52] in 1998 using the L-polylactic acid (PLLA) stent. Subsequently, great deals of scholars have conducted research on degradable polymer stents. In 2000, Tamai et al. [53] used the Igaki-Tamai-PLLA biodegradable stent for the first human trials. The stent had a thickness of 0.17 mm and a zigzag spiral shape (as shown in Figure 5a) and was implanted in 15 patients. Follow-up angiography and intravascular ultrasound examinations at three and six months after stent implanted showed that no thrombosis or major cardiac events occurred within 30 days, and no major cardiac events occurred within six months except for repeated angioplasty. Erbel et al. [54], Moravej et al. [55], and Hehrlein et al. [56] studied magnesium alloy, iron alloy, and zinc alloy degradable vascular stents (as shown in Figure 5b–d) and achieved certain research results. BDSs have great advantages over BMSs and DESs. Nevertheless, there are also a series of problems. For example, biodegradable polymer stent has defects such as poor mechanical properties, high elastic recoil, and fast mechanical attenuation [57]. The matrix of the magnesium alloy stent degrades quickly, and the radial force is obviously weak in the later stage of implantation, and the place of stent implantation is prone to late retraction [58]. Fe-alloy stent can interfere with MRI imaging, degrade unevenly, and produce residues in the body [59,60]. Zinc alloy stent is still in the preliminary stage of research, and there are defects of uneven degradation and many problems that have not been discovered yet [51]. The current methods to solve the mechanical properties and degradation problems of BDSs, in addition to develop new materials, designing structures of stents can also be considered [61,62] to improve radial stiffness, axial flexibility, and reduce foreshortening, etc. [63,64,65].

The above-mentioned vascular stents, their structures, and materials affect the mechanical properties and the manufacturing methods. From BMSs to BDSs is not only the evolution of stent material but also the evolution of structure. With the emergence of new materials and optimization of the structure, stent material has changed from stainless steel to degradable alloys and degradable biomaterials, strut thickness of stent has reduced. Generally, changing the structural design dominates the mechanical properties of the stent. However, due to the limited choice of materials, materials are usually used to improve biocompatibility. Table 1 lists materials and clinic performances of stents. Moreover, stent implantation can induce complications and affect the second operation.

### 2.4. The Delivery Devices and Methods of Stents

**Figure 6:**
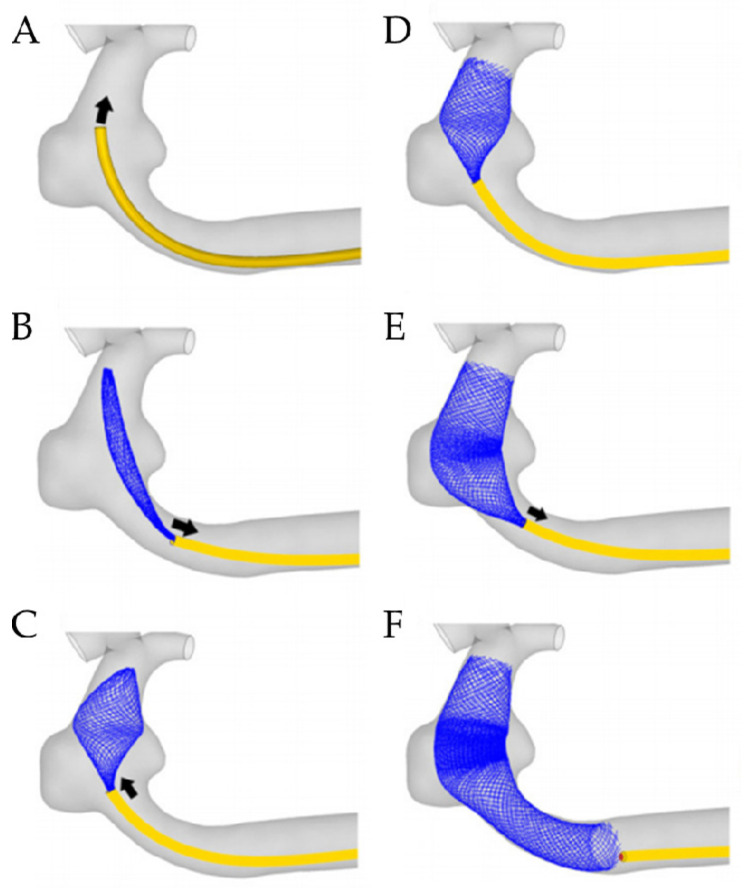
Stepwise deployment of stent [69]. (**A**) the delivery of stent; (**B**–**F**): the retraction of microcatheter and expansion of stent.

The entire deployment of the stent is a complex process, including crimping stent, fitting into a microcatheter, delivering the stent-microcatheter system, and release from the microcatheter. The purpose of all work is to accurately deploy the stent to the stenosis of a blood vessel and then release the stent to open the vessel. A microcatheter, as the delivery device, is extremely important and complex. The delivery device affects the deployment and expansion, which is an important part of therapy. Meng’s team [66,67,68,69] has been doing research on stent delivery and deployment. They have established a simulation workflow similar to the actual clinical work, which is highly reliable. When simulating the real delivery pathway, the biggest difficulty is to set the motion path of the microcatheter. Meng et al. [69] extracted a series of reference points and associated normal directions from the center of a sequence of lumen cross-sections to represent the prescribed delivery path. Figure 6 shows the delivery, deployment, and release of the stent. However, there are some drawbacks to their work. The artery wall was a rigid body in Meng’s team, which is inconsistent with a real vessel wall. Additionally, some literature [70,71,72,73,74] did research on the delivery and deployment of a stent. Babiker et al. [70] proposed different method to simulate the pathway of delivery. They extracted the nodes on the central line of the microcatheter to define the pathway by imposing boundary conditions for different nodes in a Cartesian coordinate system. Wang’s team [71] and Zhang and Xiang’s team [72] extracted the central line of the patient’s blood vessel as the pathway of delivery stent. All of these research have made some progress and simulated the crimper, delivery, deployment, and release of vascular stent approximately. It also proves that finite element analysis is an important application in medical field and has the reliability of results.

### 2.5. Introduction to the Manufacturing Method of Vascular Stents

**Figure 7:**
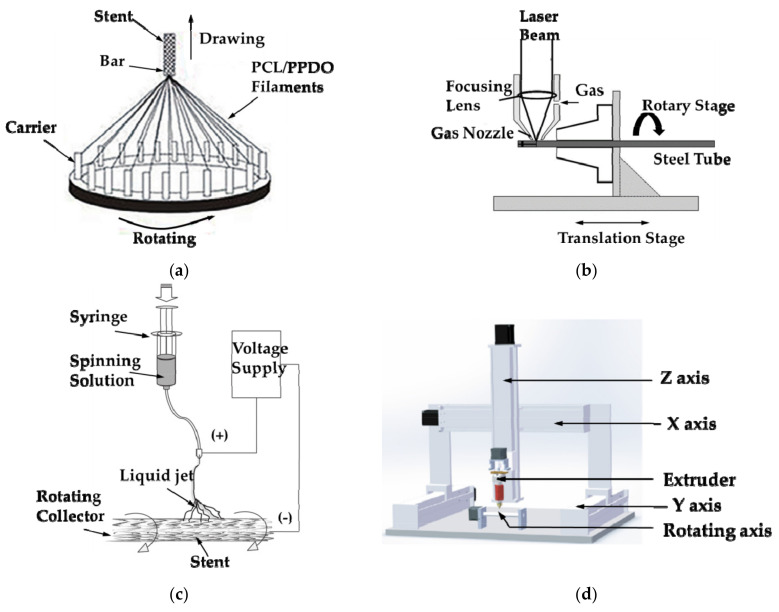
Vascular stent processing method: (**a**) Braided [77]; (**b**) Laser cutting [80]; (**c**) Electrospinning technology [86]; (**d**) Additive manufacturing [91].

As the precise medical device, the manufacturing methods of vascular stents mainly include braided method (Figure 7a) [75,76,77,78], laser cutting method (Figure 7b) [79,80,81,82], electrospinning technology (Figure 7c) [83,84,85,86] and additive manufacturing technology (Figure 7d) [87,88,89,90,91]. The braided method is to first wind the wire on the carrier. There are multiple carriers in Figure 6A, whose purpose is to use the two kinds of filaments to fabricate the stent at the same time. Draw the cylindrical bar upwards and rotate carriers at the same time, so that the filaments are wound around the bar, thereby forming a mesh-like braided structure stent. As the stent structure tends to become more complicated, the vascular stent manufactured by the braided method is limited to a simple structure, and the stent has poor radial stiffness, so the braided method is no longer suitable for fabricating stent. 

The laser cutting method mainly utilizes the high temperature generated by the laser beam to instantaneously melt the material, and the material is rotated on the machine tool so that the laser can cut along the circumference of the tubular material, and finally fabricates the stent. However, the laser cutting method limits the stent material and is mostly used for metal materials, and the heat-affected zone generated by the laser causes the surface quality of the stent to deteriorate. 

Electrospinning technology is the preparation of small-diameter vascular stents under tens of thousands of voltages, which makes high spinning solution or melt charged. When the charge reaches the critical value, the electrostatic repulsion force makes the solution overcome the surface tension to form a jet stream, and the vascular stents are obtained through the rotating collector device with different diameters. Although the electrospinning technology is simple to operate, the electrospinning technology is not suitable for preparing complex vascular stent structures and is mostly used for preparing small-caliber stents or coating metal stents, which results in limited application of electrospinning technology. 

At present, additive manufacturing technology has become mature. Most scholars mainly improve Fused Deposition Modeling (FDM) technology to fabricate vascular stents. As shown in Figure 7d, a rotating shaft is added to the printing platform, and the printing filament is melted at high temperature and sprayed through the nozzle, and deposited on the rotating shaft to prepare the stent. Because the additive manufacturing technology has the characteristics of a short printing cycle, high material utilization, complex printable structure, and various sizes of vascular stents, it has been widely used in the field of preparing stents [50]. At present, vascular stents have begun to tend to patient-specific design, and the application of additive manufacturing technology provides unlimited prospects for the design and manufacture of vascular stents.

For the currently available vascular stents, their in vivo performances and manufacturing methods are highly dependent on the applied materials and structural designs. Table 1 summarizes widely utilized materials for producing BMSs, DESs, and BDSs, as well as comparing their advantages and limitations. However, the options of suitable materials are limited because of their weak radial strength, toxic degraded products, intractable manufacturing, and so on. Therefore, the elaboration of stent structural design is essential to improve the mechanical properties, geometrical compatibility, and structural stability of stents to ensure their therapeutic functions.

## 3. Structure Design of Vascular Stents

### 3.1. Evaluation Standards of Mechanical Properties

The design of the stent structure is of great significance for the treatment of atherosclerotic blood vessels. If the stent deforms unevenly during the expansion process, it will cause serious damage to the blood vessels. In view of the above-mentioned types of vascular stents and the problem of ISR, this paper summarizes the ideal functions and mechanical properties of BMSs, DESs, BDSs [5,63,64,65,92,93]. As a result, when designing the structure of vascular stent, there are some standards to evaluate stents:

#### 3.1.1. Radial Stiffness or Radial Elastic-Recoil

The radial elastic-recoil of vascular stent refers to the expansion of the stent under the action of the balloon. After the balloon is removed, the expansion force of the balloon on the stent will disappear. At this time, because of the radial pressure of the vascular wall on the stent and the elastic deformation of the stent itself, the stent will have certain radial elastic recoil. If the stent undergoes large elastic recoil, the radial support effect of the stent on the vessel wall will be severely weakened, and ideal vascular expansion cannot be achieved. Equation (1) is the radial elastic recoil formula of the stent:

*R*_*recoil*_ = (*R*_*load*_ − *R*_*unload*_)/*R*_*load*_ (1)

In the formula, *R_load_* represents the radial diameter of the stent when the balloon is fully expanded, and *R_unload_* represents the radial diameter of the stent after the balloon is removed.

The radial elastic-recoil of the stent is related to the stiffness. Supposing that the stent has a large elastic-recoil after expanding, the stent cannot support blocked blood vessels. As a result, the stent does not strut plaques and restore blood flow.

#### 3.1.2. Foreshortening

After the vascular stent expands radially, the designed stent structure has a negative Poisson’s ratio, resulting in a certain shortening in the axial direction. If the stent is severely shortened, the stent cannot be accurately placed to the position of the blood vessel plaque, which affects the outcome of the treatment. Equation (2) is the foreshortening formula of the stent:

*Foreshortening* = (*L*_0_ − *L*_*final*_)/*L*_0_ (2)

where *L*_*0*_ is the initial axial length of the stent without any deformation, and *L_final_* is the final axial length of the stent after positioning in the blood vessel.

The foreshortening affects the accurate positioning of the stent in the vessel. When the stent is implanted into the blood vessel and placed in the location of the plaque through the catheter, if a stent causes the foreshortening, the expanded stent can’t completely cover the plaque or even deflect away from the blocked vessel under blood flowing, making it impossible to cure atherosclerosis. 

#### 3.1.3. “Dogbone”

When the stent is in the process of vascular expansion, the stent will deform unevenly under influence of a balloon, which usually shows that the two distal of the stent expand more than the middle part, forming a shape similar to a “dogbone”. Equation (3) is the “dog bone” formula:

*Dogbone* = (*r*_*distal*_ − *r*_*central*_)/*r*_*distal*_ (3)

where *r_distal_* is the radius at both ends of the stent, and *r_central_* is the radius at the middle of the stent.

The “dogbone” of stent often occurs using balloon expansion. If the stent undergoes non-uniform “dogbone” deformation, it will cause damage to the inner wall of the blood vessel and result in complications such as ISR.

#### 3.1.4. Axial Flexibility

After the stent expands in the blood vessel, the stent is not prone to bending deformation due to the restriction of the radial stiffness. As a result, it is difficult to achieve the same degree of bending between the stent and the blood vessel. It leads to poor adhesion to the inner wall of the blood vessel, resulting in high wall stress and large damage caused by the implantation of the stent [94]. The higher the radial stiffness, the greater the pressure on the vascular wall of the stent, and the better it can ensure smooth blood flow; the higher the axial compliance, the easier it is for the stent to achieve bending deformation, and the less damage to the wall of the blood vessel with a higher degree of curvature. Solving the two contradictory coexistence problems of axial flexibility and radial stiffness is an important standard when designing stents.

**Figure 8:**
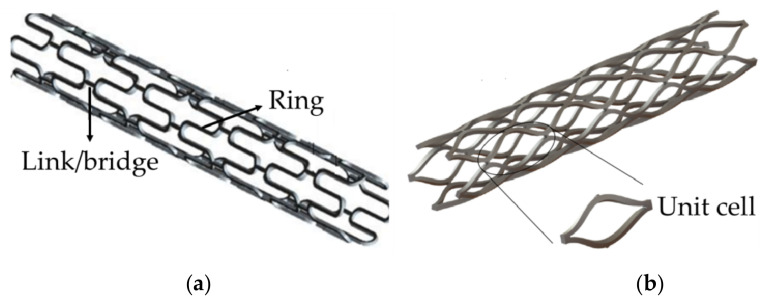
Two types of vascular stent designs: (**a**) Link/bridge stent [86]; (**b**) RVE/RUC stent [96].

Therefore, designing a stent should avoid undesired structural failures. The above-mentioned standards are important for designing stents. The structural design of vascular stent is directly related to its mechanical properties, whether it is the first generation of BMSs or DESs, as well as BDSs, all need to improve the performances of the stent in terms of structural design and optimization. In this paper, vascular stents are divided into two types. One is composed of the rings and the links (also called “bridge”) [95], as shown in Figure 8a. The function of the rings is to radially expand and support the blood vessel, and the function of the links is to connect the rings axially to achieve the axial flexibility of stents. The shape and size of bridges is usually a hot spot for scholars to research and design. The second type is a scaffold structure formed by directly connecting and arranging representative volume elements (RVE)/representative unit cell (RUC), as shown in Figure 8b [96]. By designing different unit cell structures, the stent with target deformation can be obtained, which also is a hot research content of stent design. In order to facilitate readers to understand the mechanical properties of different stents, Appendix A lists the advantages and disadvantages of each stent mentioned in this paper.

### 3.2. Design of “Bridge/Link” Stents

**Figure 9:**
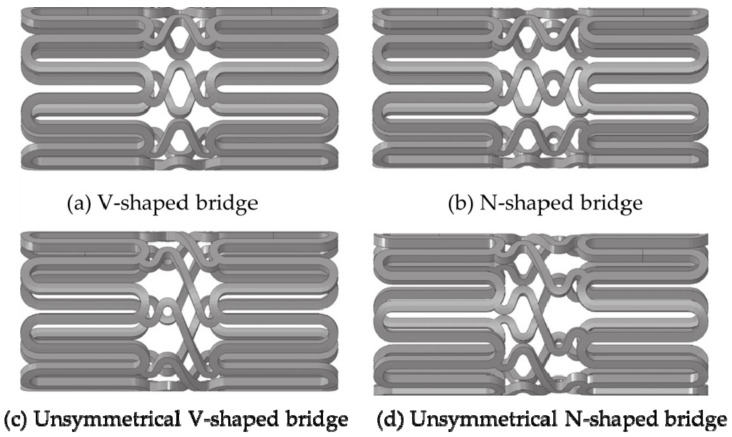
Different bridge structures [92]: (**a**) V-shaped; (**b**) N-shaped; (**c**) Unsymmetrical V-shaped; (**d**) Unsymmetrical N-shaped.

The link/bridge is one of the important factors affecting the axial flexibility of the stent [84]. Regarding the design of the “bridge” vascular stent, the researchers pointed out that the geometric parameters of the link/bridge determined the mechanical properties of the stents [92,94,97,98,99,100]. At present, the bridges of vascular stents roughly include L-shaped, N-shaped, V-shaped, S-shaped, etc. In the analysis of the mechanical properties of the vascular stents with L-shaped, V-shaped, and S-shaped, Behrend used the cantilever method to verify that the RX Multilink stent with an L-shaped bridge had the smallest axial stiffness [99]. However, due to not involved the S-shaped stent in the experiments, and the number of experimental samples was too small, the conclusion cannot be used as an evaluation standard for different bridge stents. Ormiston et al. [100] used the three-point support method to study vascular stents with a variety of bridge structures. The results showed that the performance of the S-shaped vascular stent was better than that of the L-shaped and V-shaped stents, and the axial flexibility of the L-shaped and V-shaped stents was almost the same. Similarly, Wei et al. [101] pointed out that among the six different stent structures, when the vascular curvature was 0° and 15°, the stent with an S-shaped bridge structure was the most flexible. When the vascular curvature was 30°, 45°, and 60°, the U-shaped stent had the best flexibility. However, these studies are based on the finite element simulation of the ideal model analysis, and do not simulate the effect of balloon dilatation on the structure and ignore the role of intravascular plaque. Azaouzi et al. [92] separately studied the effects of V-shaped, N-shaped, unsymmetrical V-shaped, and unsymmetrical N-shaped on the mechanical properties of balloon-expandable stents (as shown in Figure 9) and conducted finite element analysis on the axial flexibility and radial strength of stents with different bridge shapes. The results showed that in terms of bending performance, the symmetrical N-shaped bridge and unsymmetrical V-shaped bridge had better flexibility. In terms of torsional performance, symmetrical V-shaped bridge stent had the worst flexibility, and unsymmetrical N-shaped stent had the best flexibility. Since the radial force and stress of the symmetrical N-shaped bridge structure are small, it is the structure with the best radial support performance in all stents. However, the Azaouzi team do not make a quantitative analysis of the stent structures and did not directly give the radial elastic recoil rate and axial foreshortening rate of different bridge stents, but only limited to a qualitative evaluation of the structure. 

**Figure 10:**
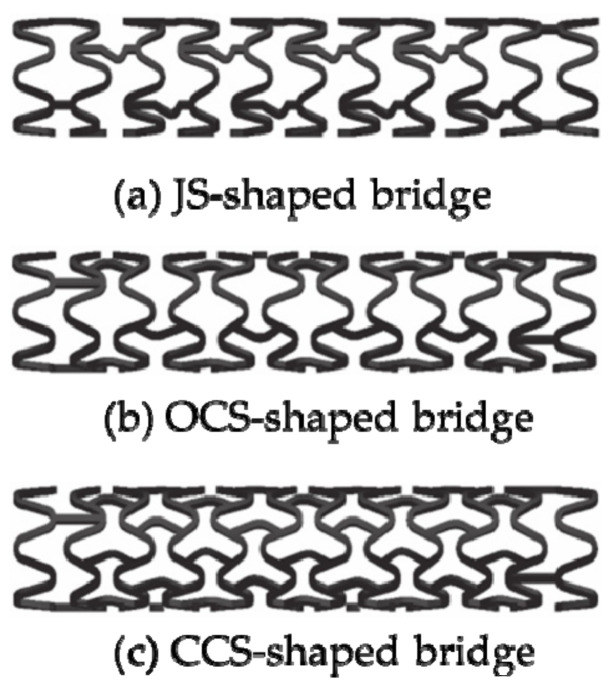
Models of vascular stents [102,103]: (**a**) JS-shaped; (**b**) OCS-shaped; (**c**) CCS-shaped.

**Figure 11:**
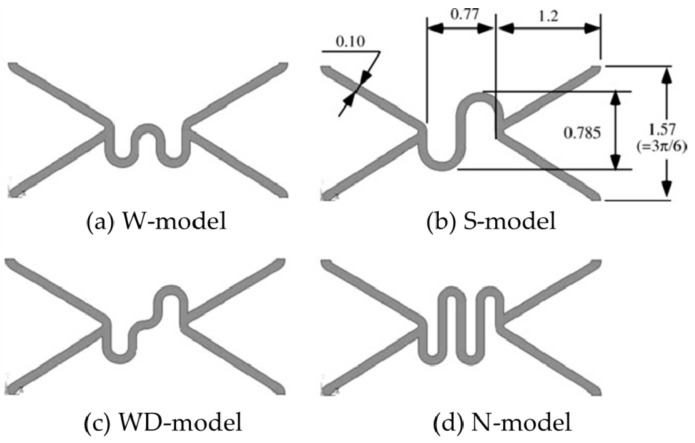
Four different model structures [104]: (**a**) W-model; (**b**) S-model; (**c**) WD-model; (**d**) N-model.

Wei et al. [102,103] designed the JS-shaped bridge, the OCS-shaped bridge, and the CCS-shaped bridge (as shown in Figure 10), and used the plane compression method, the V-groove compression method, and the three-point bending method to study the mechanical properties of stents. The experimental results showed that the radial strength of the JS-shaped stent, the open OCS-shaped stent, and the closed CCS-shaped stent was 14%, 34%, and 42% higher than the radial strength of the ordinary biodegradable stent structure, respectively. The bending stiffness of the JS-shaped and OCS-shaped was equivalent to that of the ordinary biodegradable stent structure, which was reduced by about 73% compared with the CCS-shaped. All stents had no axial foreshortening. Although the radial stiffness has been improved, they do not take into account the radial stiffness and axial flexibility performance when designing stents with various bridges. For the study of the axial flexibility of vascular stents, Mori and Saito [104] analyzed the influence of four different structures, W-shaped, S-shaped, WD-shaped, and N-shaped on the compliance performance of stents (as shown in Figure 11). They used the four-point bending test method to test the bending stiffness of the stents and found that the S-shaped stent was 85.28 N mm^2^, the N-shaped stent was 41.67 N mm^2^, and the improved WD-shaped stent was 78.79 N mm^2^, W-shaped stent was 188.67 N mm^2^, respectively. This test provides a new method for studying the axial compliance and flexibility of vascular stents, namely the four-point bending test. Similarly, these studies have not balanced the radial stiffness and axial compliance, and they are all based on a single factor to design the bridge to improve the structure of the stent.

**Figure 12:**
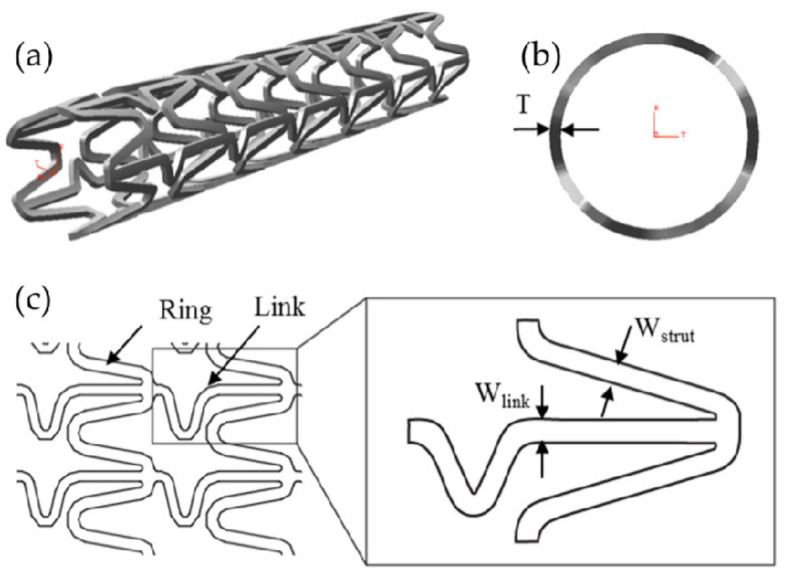
MAC-Plus stent and parameters [105]: (**a**) Oblique view of MAC-Plus; (**b**) View along z-axis (axial direction) is used to show the stent thickness; (**c**) Planar view of the MAC-Plus stent structure.

In order to obtain better mechanical properties and design a better stent structure, researchers optimized the bridge structure of vascular stent, and studied the influence on the mechanical properties by changing the length and width of the bridge. Tammareddi et al. [105] adopted a controlling variables approach to aiming at multiple optimization goals such as increasing radial stiffness, improving axial flexibility, and reducing the maximum stress on the vessel wall (as shown in Figure 12), and analyzed 4 × 23 sets of stents with different geometric parameters by means of finite element simulation. The research results indicated that by reasonably reducing the width of the bridge *W_link_* and increasing the length of the bridge *L_link_* could appropriately improve the axial compliance and flexibility of vascular stent and reduce the maximum stress on the vascular wall, but this would also cause the stent had an excessive axial foreshortening during expansion, which would affect the precise positioning and restore the diseased blood vessel. Wang’s team [106] used finite element simulation technology to analyze six sets of stents with different bridge widths and concluded that appropriately increasing the width of the bridge could effectively reduce the “dogbone” deformation of stents after expanding. However, due to the discrete comparison of the differences between different designs, this study does not give the optimal design.

### 3.3. Design of RUC/RVE Stents

**Figure 13:**
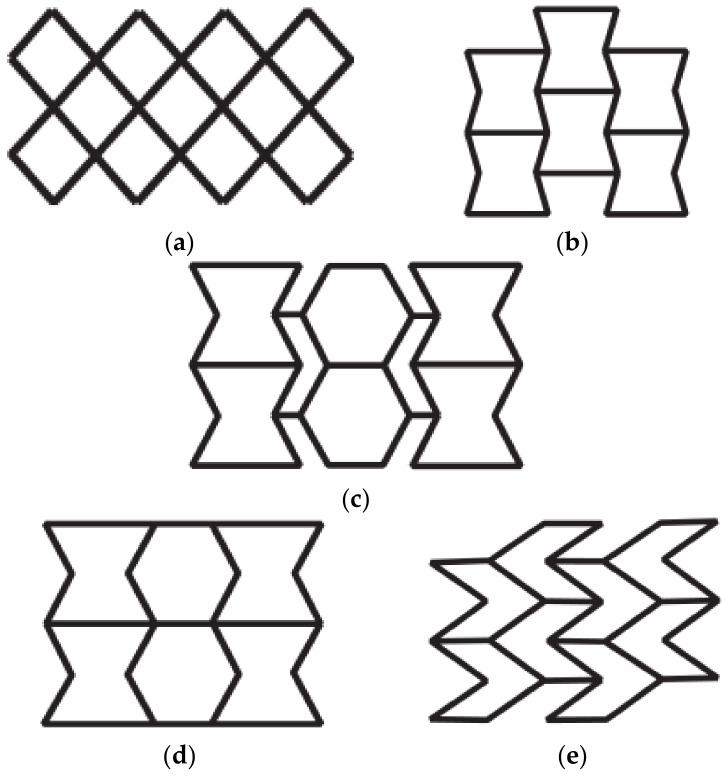
RVE structure of vascular stent [96]: (**a**) Diamond; (**b**) Reentrant Auxetic; (**c**) Hybrid A; (**d**) Hybrid C; (**e**) Chevron B.

**Figure 14:**
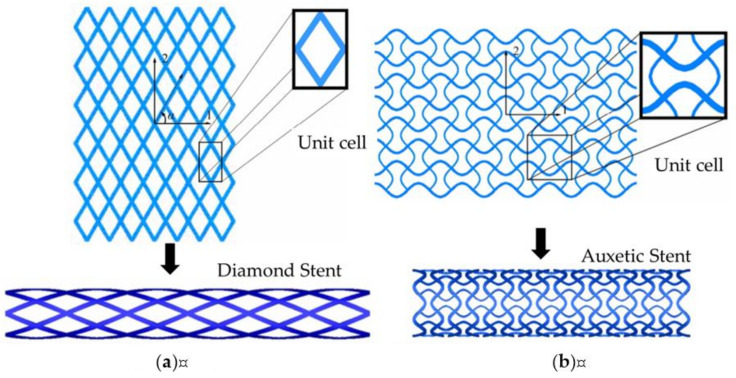
Two-unit cell of stents [110]: (**a**) Diamond stent; (**b**) Auxetic stent.

In the structural design of vascular stents, the representative volume element (RVE)/representative unit cell (RUC) is often studied [107,108]. By designing the unit cell and analyzing its performance, the design efficiency is improved, and the diversification of the stent structure can be realized. The Prithipaul team [96] designed vascular stents with different RVE structures (as shown in Figure 13) and compared the mechanical properties. They carried out an experimental analysis of mechanical properties from the radial elastic recoil, foreshortening, radial stiffness, and Wall Shear Stress (WSS). They deemed that except the Diamond structure exhibiting poor mechanical properties, Reentrant Auxetic, Hybrid A, Hybrid C, and Chevron B exhibited better radial stiffness, foreshortening respectively. Each structure cannot have the advantages of multiple mechanical properties. For example, the radial stiffness and WSS of the Diamond stent were contradictory. Douglas et al. [109] also studied five-unit structures of vascular stents Diamond, Reentrant Auxetic, Hybrid A, Hybrid C, and Chevron B, and their conclusions were consistent with Prithipaul et al. In addition, Dolla et al. [110] and Tan et al. [111] both verified the conclusion that the Reentrant-Auxetic stent had good mechanical properties. Figure 14 is two vascular stents with different unit cells designed by Dolla et al. [110].

**Figure 15:**
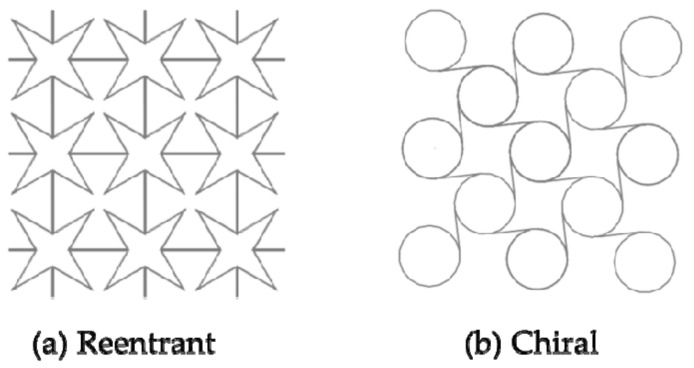
Auxetic structures of application stents [115]: (**a**) Reentrant; (**b**) Chiral.

**Figure 16:**
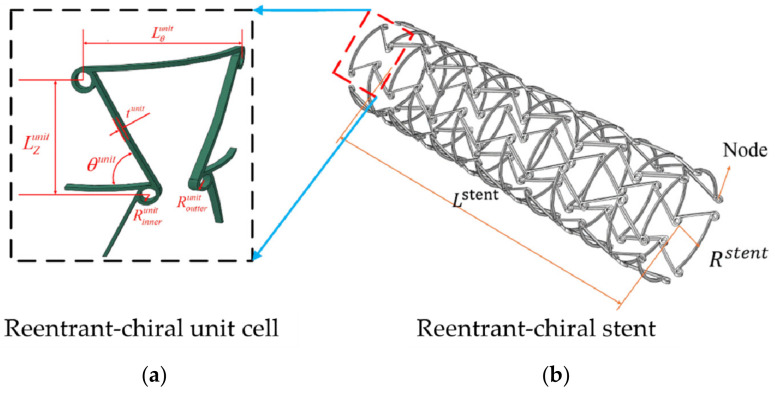
Reentrant-chiral stent [117,118]. (**a**) Reentrant-chiral unit cell, (**b**) Reentrant-chiral stent.

The auxetic structure is the negative Poisson’s ratio material that expands/contractions during tension/compression [112]. This special deformation behavior produces some favorable mechanical properties, such as excellent resistance to indentation, resistance to shear and fracture resistance, enhanced sound absorption, variable permeability, etc. [113]. In view of these properties, Auxetic structure has been used in the design of self-expanding vascular stents [114]. Reentrant (Figure 15a) and Chiral (Figure 15b) are Auxetic structures currently used for vascular stent research [115]. Liu et al. [116] designed the Reentrant shape memory polymer vascular stent. Numerical analysis results indicated that the Reentrant stent with a smaller radius had a higher critical buckling load and a smaller buckling displacement. Compared with traditional stents, the contact area between the stent and the blood vessel was smaller, the stress was smaller after implantation. And they explained that the radial strength depended on the stent radius and the number of circumferential unit cells. Based on the Reentrant structure and the Chiral structure, the Ruan team [117,118] designed the Antichiral-Reentrant vascular stent (Figure 16). They verified that the Antichiral-Reentrant stent had good mechanical properties after being implanted in the blocked lesion by designing stents of different sizes. But for the research of Antichiral-Reentrant stent, during the finite element analysis, the Ruan team still set the balloon as a rigid surface, which did not match the actual clinical balloon expansion method, and did not explain radial stiffness, foreshortening, “dogbone” of stents.

**Figure 17:**
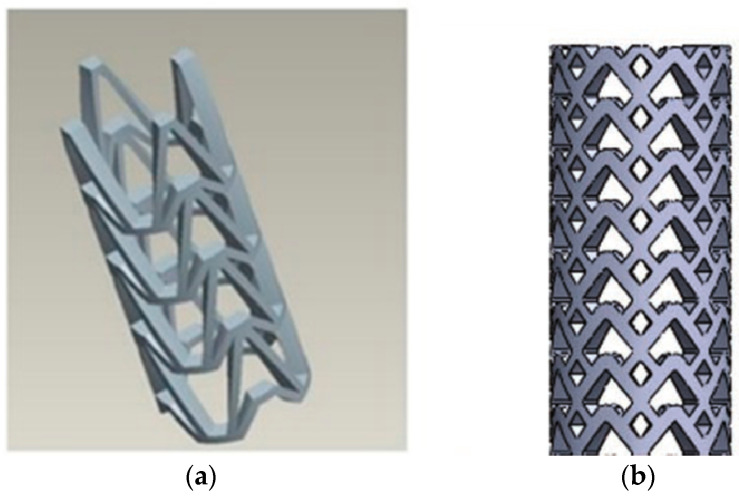
Arrowed stent: (**a**) Arrowed stent of Wu [119]; (**b**) Arrowed stent of Ameer [120,121,122].

Currently, for the structure design of vascular stent, in addition to the introduced Auxetic structures such as Reentrant, Chiral, and Reentrant-chiral with negative Poisson’s ratio, scholars have designed the Arrowed stent. However, the Arrowed stent is still in the initial stage of research, and its mechanical properties have not been rigorously scientifically studied. The Arrowed stent manufactured by Wu et al. [119] using FDM technology (as shown in Figure 17a) was of poor quality and required a long time of post-processing. The Ameer team [120,121,122] synthesized a new Arrowed degradable polymer stent (Figure 17b), and successfully achieved non-supported printing by using a new micro-continuous liquid interface manufacturing technology. This research reduced the cumbersome procedures for post-processing of vascular stents, and the Arrowed stents had good elasticity, high strength, anti-oxidation, and biodegradability.

**Figure 18:**
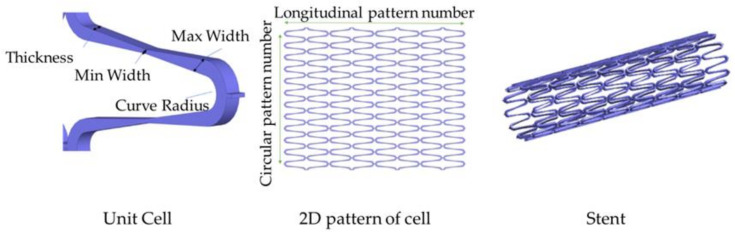
Non-uniform size of stent [123].

In addition to the stent design with uniform cell size, Torki et al. [123] designed a stent structure with a non-uniform cell size (Figure 18). They optimized the parameters to obtain the best stent model and placed the optimized stent in an artery with 49% plaque unevenness to simulate the performances of the stent. The finite element results showed that the internal area of the artery cross-section increased 74%, which meant that blood flow had improved by 74%. The concept of designing and optimizing the non-uniform stent proposed by Torki et al. has great significance for designing structures of stents, which provides new ideas for improving the function of stents.

### 3.4. Design of Patient-Specific Stents

**Figure 19:**
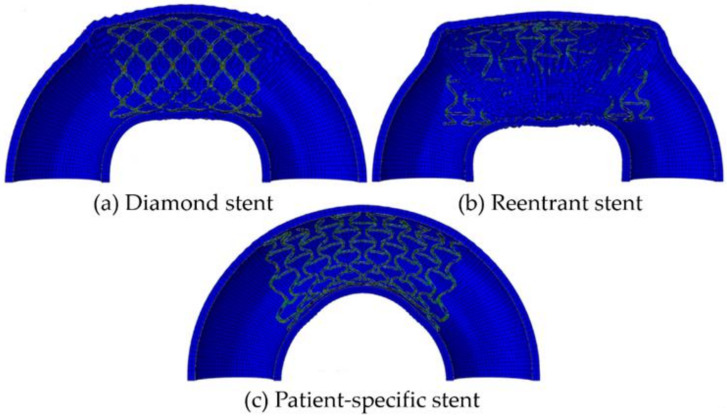
FEA results of four different stent-vessel systems after expansion [124]: (**a**) Diamond stent; (**b**) Reentrant stent; (**c**) Patient-specific stent.

**Figure 20:**
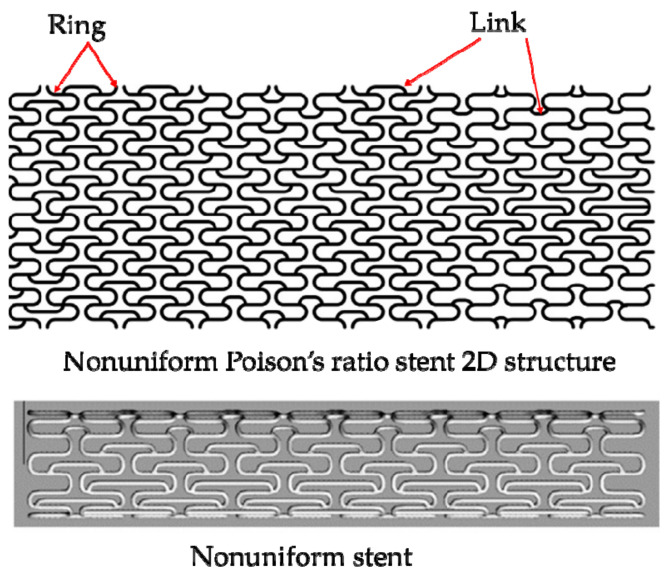
Nonuniform stent [124].

**Figure 21:**
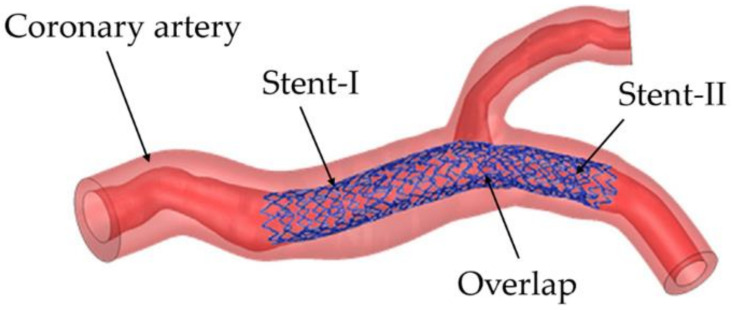
Implantation of two stents facing the target vessel [126].

At present, the realization of patient-specific vascular stents for patients with vascular disease is becoming a research hotspot. Designing the patient-specific stent relays on the shape of the patient’s blood vessel. The patient-specific stent is able to achieve an identical shape as the blood vessel after deployment and deformation. This geometrical match can avoid or significantly reduce the interaction stress between the stent and vascular wall after; and thus, can effectively reduce ISR. That is because two ends of the patient-specific stent do not trigger stress concentration in a vessel and can be in compliance with the shape of the vessel. Adversely, conventional stents straighten the vessel resulting in stress concentration, which triggers complications such as ISR. Consequently, scholars focus on designing the patient-specific stent. Han and Lu [124] designed a vascular stent with non-uniform Poisson’s ratio for patients with curved blood vessels and performed a finite element comparative analysis with Diamond stents and Reentrant stents with uniform Poisson’s ratio (Figure 19). Diamond stent and reentrant stent do not exhibit the same curvature as the blood vessel after expansion. The two ends of the stent cause severe stress concentration on the vessel wall, resulting in vascular intimal hyperplasia and high ISR. But the non-uniform Poisson’s ratio vascular stent designed by Han and Lu can have the same curvature as a curved blood vessel, flexible in axial, and does not cause stress concentration on the inner wall of the blood vessel. Han and Lu pointed out that by adjusting the position of the connecting place between the link and the ring, that is, changing the length of the link *L_link_* (Figure 20), the Poisson’s ratio of the unit cell can be changed from positive to negative. According to the performance of the non-uniform Poisson’s ratio structure, Han and Lu designed a patient-specific stent for the patient’s blood vessel. In fact, before Han and Lu, Auricchio et al. [125] had already researched target vascular stents for patients, but the blood vessels they studied were blood vessels with a small degree of curvature, and they did not solve the complicated design of vascular stents. Morlacchi et al. [126] tried to study the stent of the target blood vessel, but for the diseased part of the curved blood vessel, they adopted the method of implanting two vascular stents, and the two stents overlapped at the curved part (as shown in Figure 21). Such the structure does not allow the stent to fit the blood vessel perfectly. On the contrary, it still causes stress concentration on the inner wall of the blood vessel at both ends of the stent. When Ragkousis et al. [127] solved the problem of target vessel stenosis, they only evaluated the existing stent structure. Although the stent structure was optimized, the blood vessels they studied have low curvature and lack research on axial compliance performance.

## 4. Perspectives on the Future and Challenges of Designing and Fabricating Stents

At present, cardiovascular disease has become the primary cause of death in humans. Although PCI can alleviate the patient’s bad condition, there are still many problems, such as ISR. Therefore, a reasonable structure of the stent can effectively solve the complications after implanting it into the blood vessels. Faced with such problems, whether it is BMSs, DESs, or BDSs, it is necessary to study the structural influence on the mechanical properties of the vascular stent. Because a first-class stent structure can better solve the contradiction between “radial stiffness” and “axial flexibility”, it can not only ensure the radial stiffness but also improve the axial flexibility of the stent. In addition, an outstanding stent structure can also increase the drug loading rate of the DESs and balance the degradation rate of the BDSs. At present, the existing structures of stents have different characteristics. However, there are few stents with multiple properties at the same time. The performance of different types of stents has been summarized, see Appendix A. 

In the future of vascular stents, research should mainly focus on the key issues of high stress, high damage, and high restenosis rate after the stent implantation, especially curved blood vessels. More importantly, different patients have different degrees of vascular stenosis. Therefore, vascular stents tend to be patient-specific and customized for patients, so that cardiovascular diseases such as atherosclerosis can be effectively treated, and the damage caused to patients after the implantation of existing structural stents is avoided. The structure design of stents is one of the important methods to realize patient-specific stents. As mentioned in this review, a reasonable structure can effectively improve the characteristics of the stent.

Additionally, delivery and deployment of the stent is extremely important. It is a very complicated process, whether it is a simulation or clinical operation. Accurate delivery and deployment are necessary for the treatment of atherosclerosis. The release of the vascular stent in the prescribed position is a necessary condition for precision medicine and patient specific. Therefore, researchers cannot ignore any part of the crimper, delivery, deployment, and release of stents. In future work, we need to evaluate the mechanical properties of vascular stents more comprehensively.

Besides, fabrications of the stent should also not be limited to current manufacturing methods. It is difficult to fabricate vascular stents with complex structures and precise size with the existing methods. Therefore, it is necessary to improve the processing method for the fabrication of stents. Stereolithography (SLA) is an additive manufacturing technology with extremely high printing accuracy, which can be used to fabricate stents, for example, Ameer’s team [120,121,122] successfully used SLA technology to achieve unsupported printing by designing an arrow-shaped stent. The fabricating method will have a direct impact on the performance of stents. Therefore, comprehensive consideration of the structure and the manufacturing methods of stents can make medical devices have a broader application field.

Finally, the combination of medical and structural design may improve the real role of vascular stents. Scholars in the field of structural design should communicate with cardiovascular experts in hospitals so that the design and manufacture of vascular stents can meet the needs of hospitals and patients. This can not only greatly promote the design and manufacture of stents, but also enable hospitals to treat patients and save lives. At the same time, it is also an important premise to realize the patient-specific stents.

## 5. Conclusions

This paper summarizes the various structures of a vascular stent, briefly expounds on the development process of the stent, as well as the criteria for evaluating the mechanical properties, delivery and deployment, and the manufacturing method of a stent. This paper focuses on the structural design of the stent from the three types including the bridge stent and the RUC/RVE stent, and patient-specific stent. We analyze the problems and shortcomings of the existing stent structure. It is pointed out that the stent structure is designed simply, and it is difficult to balance the radial stiffness and axial compliance. Additionally, current research on vascular stents is still limited to improved and optimized design on the existing stent structure and lacks patient-specific designs for different patients.

There are also many advantages here. With the design and optimization of a vascular stent, strut thickness of stent has been substantially reduced, which is conductive to reduce injury and metal to artery ratio for BMSs. With the emergence of new materials, stent material has changed from stainless steel to degradable alloys and degradable biomaterials. This also reduces the size of the stent and the interference of BMSs on the MRI. In any case, vascular stents should be scientifically and rigorously designed and developed to ensure that the stent can effectively treat blocked blood vessels, open up thrombi, and restore vascular functions. And the design, manufacture, and engineering & medicine of vascular stents are prospected. All in all, the design of vascular stent should be in the direction of patient-specific.

## Figures and Tables

**Table 1:** Materials and properties of stents.

Therapeutic Techniques	Bare Metal Stent (BMS)	Drug Elution Stent (DES)	Biodegradable Stent (BDS)
Main materials	Stainless steel; NiTi alloy.	*Coated*: sirolimus; paclitaxel; everolimus. *The main substrates*: stainless steel; cobalt-chromium alloy; NiTi shape memory alloy.	Polylactic acid; poly-L-lactic acid; polycaprolactone; Racemic polylactic acid; Mg alloy; Fe alloy; Zn alloy.
Material strength	High	High	Moderate/low
Biocompatability	No	No/coating material Yes	Yes
Biodegradability	No	No/coating material Yes	Yes
Post-implantation Drug administration	No	Always	Always
Vascular function interruption	Yes	Yes	Yes
Incidence complicaions	High	Moderate	Low (but may be higher than DESs)
Manufacturing method	Laser cutting; Traditional cutting.	3D printing; electrospinning technology.	3D printing; electrospinning technology; laser cutting.

## Data Availability

None.

## Appendix A

**Table A1:** Comparison of the Advantages of Vascular Stents.

Types of Stent	Advantages	Disadvantages
L-shaped bridge		Axial stiffness and flexibility [99]
N-shaped bridge		Axial flexibility [92]
Un/symmetrical N-shaped bridge	Axial flexibility [92]	
S-shaped bridge	Axial flexibility [101]	
symmetrical V-shaped bridge stent		Torsional performance [92]
unsymmetrical V-shaped bridge	Axial flexibility [92]	
W-shaped bridge	Bending stiffness [104]	
WD-shaped bridge		Axial flexibility [104]
JS-shaped bridge	Bending stiffness No axial foreshortening [102,103]	Radial strength [102,103]
CCS-shaped bridge	Radial strength No axial foreshortening [102,103]	
OCS-shaped bridge	Radial strength Bending stiffness No axial foreshortening [102,103]	
Diamond		Axial flexibility Axial foreshortening Radial recoil Radial stiffness [96,110,124]
Auxetic	Radial stiffness Elastic recoil No axial foreshortening [110,111,115]	
Hybrid A	Radial stiffness Elastic recoil No axial foreshortening [96]	
Hybrid B	Elastic recoil No axial foreshortening [96]	Radial stiffness [96]
Chevron	Elastic recoil No axial foreshortening [96]	Radial stiffness [96]
Non-uniform Poisson’s ratio stent	Axial flexibility Radial stiffness Elastic recoil [124] No axial foreshortening	
